# Data Freshness and End-to-End Delay in Cross-Layer Two-Tier Linear IoT Networks

**DOI:** 10.3390/s22239455

**Published:** 2022-12-03

**Authors:** Imane Cheikh, Essaid Sabir, Rachid Aouami, Sébastien Roy, Mohamed Sadik

**Affiliations:** 1 NEST Research Group, LRI Lab, ENSEM, Hassan II University of Casablanca, Casablanca 20000, Morocco; 2 Department of Computer Science, University of Quebec at Montreal, Montreal, QC H2L 2C4, Canada; 3 Department of Electrical and Computer Engineering, University of Sherbrooke, Sherbrooke, QC J1K 2R1, Canada

**Keywords:** ad hoc network, age of information, cellular network, delay, IoT, multi-RATs integration, queuing theory

## Abstract

The operational and technological structures of radio access networks have undergone tremendous changes in recent years. A displacement of priority from capacity–coverage optimization (to ensure data freshness) has emerged. Multiple radio access technology (multi-RAT) is a solution that addresses the exponential growth of traffic demands, providing degrees of freedom in meeting various performance goals, including energy efficiencies in IoT networks. The purpose of the present study was to investigate the possibility of leveraging multi-RAT to reduce each user’s transmission delay while preserving the requisite quality of service (QoS) and maintaining the freshness of the received information via the age of information (AoI) metric. First, we investigated the coordination between a multi-hop network and a cellular network. Each IoT device served as an information source that generated packets (transmitting them toward the base station) and a relay (for packets generated upstream). We created a queuing system that included the network and MAC layers. We propose a framework comprised of various models and tools for forecasting network performances in terms of the end-to-end delay of ongoing flows and AoI. Finally, to highlight the benefits of our framework, we performed comprehensive simulations. In discussing these numerical results, insights regarding various aspects and metrics (parameter tuning, expected QoS, and performance) are made apparent.

## 1. Introduction

One recent significant advancement of the information age is the Internet of Things (IoT), which provides convenient benefits, resulting in the widespread growth of mobile network services and the promotion of more comfortable and relevant lifestyles and facilities. However, this rapid development has resulted in a large rise in energy consumption, leading to greater greenhouse gas emissions and higher financial expenses for network operators. Energy costs associated with the operation of a cellular network now account for a sizable share of the global human energy footprint. As a result, network operators are searching for innovative ways to reduce and manage their energy footprints [1]. Overall, for a sensor network without energy recovery capabilities, energy-efficient communication technology is required for data transmission. A sensor will be unusable immediately after its battery is discharged (if no alternate power source is available). Therefore, it is crucial to understand and characterize the performances of sensor networks, especially in terms of delay and energy consumption. Ideally, a sensor network should have the longest operating life before requiring maintenance (such as a battery change). Consequently, it is necessary to operate such networks at the lowest possible energy consumption; this has been an ongoing area of research [2].

To address these issues, the development of advanced wireless systems and services is taking place in a heterogeneous environment where multiple RATs coexist. As a result, the complexity and cost of network deployment decrease, leading to even higher energy efficiency gains.

Currently, different radio access technologies (RATs) typically operate independently from each other. However, there is a growing demand for coordination between different RATs to meet the exponential growth in wireless traffic. Mobile users or autonomous sensor nodes can be served simultaneously by two or more RATs. Commonly, multiple connections participate in the application or transport layer, and each connection (or flow) corresponds to a single RAT (5G, LoRa, NB-IoT, LTE-M, etc.) over which the data stream [3]. The collaboration enables and maintains connectivity for universal use and provides the most appropriate services for users, regardless of time or location. With multiple radio interfaces, IoT devices are granted the ability to communicate simultaneously over different interfaces and select the “best” interface at any given moment based on a variety of parameters, such as QoS requirements, network capabilities, application properties, etc. Essentially, for each interface, there is a specific range and cost (energy, economic issues, etc.) [4].

Keeping all of these considerations in mind, the goal of this article is to address the minimization of the total delay in a multi-RAT network while taking into account data freshness. The integration of a multi-hop wireless ad hoc network and a cellular network to form a multi-RAT IoT platform constitutes the core of this paper. In such a platform, the nodes coordinate and dynamically switch between RATs, with the aim of determining the best path to the destination while ensuring the data freshness and QoS constraints are met. Furthermore, multi-hop relay technology, which is widely utilized in ad hoc networks, can also advantageously be applied to cellular networks to increase network capacity [5].

A multi-hop wireless ad hoc network consists mainly of a series of nodes communicating with each other when no centralized control and fixed infrastructure are available. Many different factors, such as the routing protocol and channel access methods, play a role in making communications possible. Wireless ad hoc networks are commonly used for commercial purposes, such as providing internet connectivity to nodes that are outside the transmission range of a wireless access point. This suggests that cellular and ad hoc networks are in many ways complementary [4]. Many studies to date have concentrated on increasing network throughput and investigating the effect of modulation order on energy efficiency. In contrast, the integration of a multi-hop wireless ad hoc network with a multi-RAT system has not been investigated on the same scale. The goal of multi-RAT optimization is to discover the collection of network components that uses the least amount of energy while maintaining network QoS criteria.

A new metric known as AoI has recently been developed to quantify the freshness of information in numerous IoT applications, such as remote monitoring applications, where information has a higher value when it is fresher [6]. From this vantage point, it appears that standard performance indicators, such as packet delay and throughput, are inadequate to accurately capture the timeliness of status information based on destination data. Blindly minimizing delay or increasing throughput, for example, may not keep status information at the destination as up-to-date as possible. Hence, relying on an explicit metric such as AoI is a proper avenue for assessing the freshness of information. It is most commonly defined as the time that has elapsed since the last status packet was received at the destination, allowing source nodes to assess the freshness of information from the destination side [7].

Aside from evaluating various queuing models and policies, we are interested in identifying and understanding alternative age optimization schemes for various queues. This research also looks at the age metric when a deadline is imposed on data packets waiting in queues, forcing their removal from the system after the deadline expires. Using a deadline that is too short results in more packets expiring, resulting in fewer status updates and a higher average age. However, a deadline that is too lengthy does not remove packets that have become very stale from the queue, resulting in wasteful usage of several resources for older packets, and eventually also a rise in the average age.

### 1.1. Related Work

To fully leverage multiple networks, the multi-RAT scheme has been introduced, where multiple technologies are deployed and help users deliver services appropriately. This is a promising approach that has recently received significant attention from researchers. Many publications have been devoted to the coexistence of converged and coordinated multiple RATs, in order to reduce overall network deployment complexity and costs while improving network operations maintenance requirements. Future networks are expected to support more intelligent management and integrate a range of wireless access technologies, as well as provide some degree of self-configuration, self-optimization, and self-healing [8].

Previous research in this field has mostly focused on maximizing network capacity while adhering to QoS limitations. Other research has concentrated on the resource allocation issue for parallel transmission employing several RATs [9,10]. However, the influence of delay on system performance was not included in these contributions. The fundamental issue that must be addressed is energy consumption in wireless communications. As a result, there is a rising emphasis in a range of studies on the design of energy-efficient wireless communication systems. Based on a realistic battery model, the authors of [11] present effective relay selection and energy allocation algorithms. In [12], the authors address the problem of optimal relay and RAT selection to optimize energy efficiency. Meanwhile, an energy-efficient joint radio resource management in heterogeneous multi-RAT networks is provided in [13].

Many researchers have long been interested in the capacity analysis of wireless communication networks. As far as we are aware, no current study on multi-RAT networks has examined QoS requirements in terms of throughput, reliability, end-to-end delay, and information age, while combining cellular/ad hoc network metrics and OSI model layers. Table 1 shows a comparison of the related literature and our work. Furthermore, the effect of heterogeneous networks on the age of information has not been thoroughly or appropriately investigated so far. In contrast, extensive work has been conducted on the study of ad hoc network performance metrics while taking OSI model parameters into account. In this context, the authors of [14] analyzed the end-to-end throughput behavior and stability of transmission queues in multi-hop wireless networks. Routing, random access in the MAC layer, and topology are all taken into account in their proposed model. They demonstrated that when the queues are stable, the end-to-end throughput of a given route is not impacted by a load of intermediate nodes. In [15], the authors started from the model used in [14] and studied the interaction between the PHY, MAC, and network layers. Subsequently, in [16], the authors investigated the end-to-end performance of a multi-hop wireless network for a real-time application, based on a cross-layer scheme, including the PHY, MAC, and network layers.

As mentioned in the introduction, the AoI metric has emerged as a means to assess the quality of status updates across a wireless network. According to the authors in [17], the AoI grows until a more current status update arrives at the receiver, where successful reception entails an abrupt reduction. Such a tool is applicable in applications where the maintenance of current information is crucial. Obviously enough, the time taken to propagate through the network contributes to the degree of staleness of the received updates. As a result, adequate AoI performance is achieved when status updates are provided, not only on a regular but also timely basis. The authors of [18] discuss the age minimization issue in a multi-hop network with a broad interference restriction. Among the most relevant works, authors of [19] demonstrate that the AoI may be decreased by ensuring that newer information constantly replaces older information in the transmission queue. In [20], this concept is expanded to a multi-hop scenario. The authors of [21] outline generic AoI analysis methods, then apply these AoI approaches to a variety of increasingly more complex systems, such as energy harvesting sensors broadcasting over noisy channels, parallel server systems, and queuing networks.

Many studies have been conducted on systems with time-constrained packets. The majority of them deal with the challenge of scheduling packets in order to reduce the number of packets that expire before successful transmission. When employed in the context of a wireless sensor network (WSN), deadlines have been adopted to reduce delay and energy consumption [22]. However, few publications have investigated the deployment of deadlines from the standpoint of AoI control (e.g., [23,24]).

In conclusion, we note that the current literature on AoI has focused on many distinct types of queues, each with a particular arrival and departure procedure, queue capacity, and the number of servers.

**Table 1:** Comparison between our proposal and related work.

Reference	Topology	Performance Metrics	Main Objective	Relevant OSI Layers
[25]	Non-linear	E2E delay	To minimize the stringent task service delays for sensor and IoT devices, an analytical model was designed.	Network
[26]	Non-linear	Throughput, delay and Energy consumption	Provide hybrid HetNet offloading while taking into account user traffic loads by modeling the queues of each network user.	Network
[27]	Non-linear	E2E delay	In this paper, an analytical approach for determining the E2E mean response time of infrastructure network slices is proposed.	Network
[28]	Non-linear	Throughput, delay and Energy consumption	The development of a framework for analyzing efficient forwarding choices in terms of QoS parameters.	MAC/Network
**Our work**	Linear	E2E delay and AoI	Analyze the integration of a multi-hop wireless ad hoc network with a multi-RAT platform to optimize the energy consumption of the entire proposed system.	MAC/Network

### 1.2. Our Main Contributions

**Figure 1:**
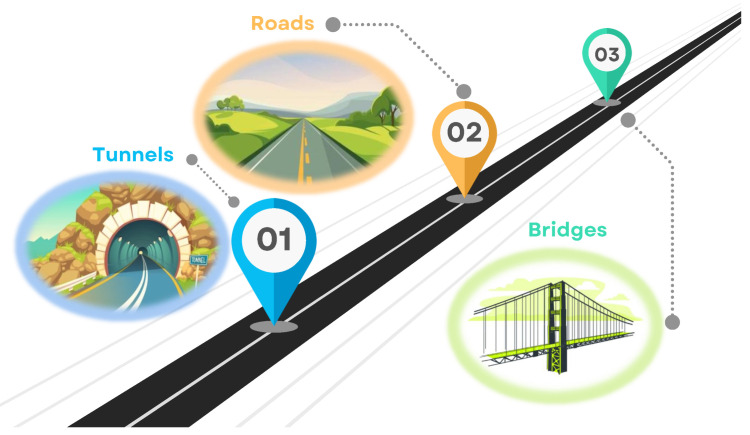
Use cases covered by our model.

The core contribution of this paper centers on the elaboration of a theoretical framework for the performance evaluation of a dual-RAT or two-tier network. Tier 1 consists of an ad hoc multi-hop network relying on a short-range, low-power, and low-cost RAT (possibly in an unlicensed band) such as Zigbee. Tier 2 consists of a centralized single-hop network with a star topology and relies on a longer range RAT, such as cellular 5G, LTE-M, LoRa, etc. Although other RAT options are possible as noted, the tier 2 connections will be henceforth referred to as “cellular”. All nodes are members of both networks and are equipped with both RATs. The physical topology of the network is assumed to be quasi-linear (as this corresponds to many applications of interest), with the base station or data sink at one end of the chain. More specifically, a probabilistic model is developed allowing us to jointly address the ad hoc/cellular channel properties and the cross-layer modeling. Our contribution can be summarized as follows:

- We build a complete framework to analyze the integration of a multi-hop wireless ad hoc network with a multi-RAT platform to optimize the energy consumption of the entire proposed system through delay minimization while ensuring data freshness, through the AoI metric.
- As illustrated in Figure 1, our model can be used in different environments such as tunnels, roads, bridges, etc.
- A cross-layer model is used, to replace the non-communicating layers of the OSI standard, involving synergy between network and MAC layers enabling the protocol stack to share specific information.
- We use a G/G/1 and an M/G/1 queuing model to estimate the waiting time at intermediate nodes.
- We determine the optimal average end-to-end delay and age of information. These two key QoS metrics provide interesting insights on how to define the internal parameters, thus achieving optimal performance.

### 1.3. Paper Organization

The rest of this paper is organized as follows. Problem formulation is discussed in Section 2, average delay analysis is defined in Section 3, the steady state and expressions for performance metrics are derived in Section 4, while the performance evaluation is addressed in Section 5. Finally, the concluding remarks and future works are presented in Section 6.

## 2. Problem Formulation

In this section, we investigate the system model, including the network topology, channel model, NET/MAC cross-layer models, energy limitations, and the proposed two-tier network incorporating both multi-hop and multi-RAT aspects.

### 2.1. The Setting

**Figure 2:**
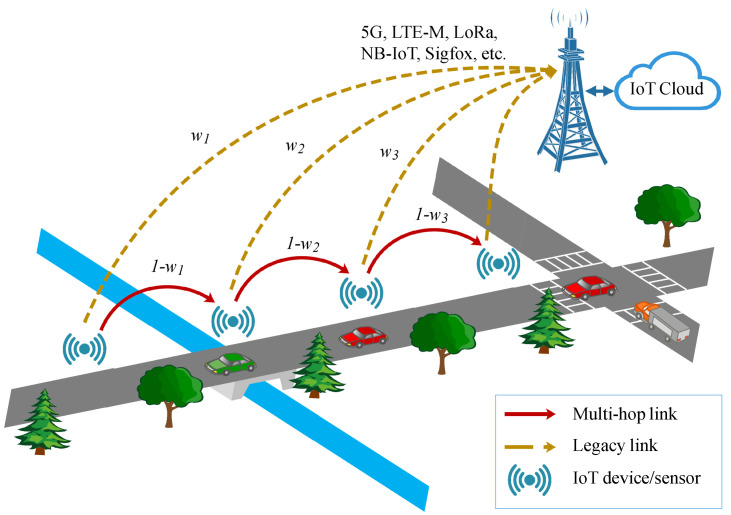
A two-tier IoT Network.

We consider a two-tier IoT network, including a base station and a set of N={1,2,3,…,n} IoT devices (such as sensors measuring temperature, pressure, vehicular speed, etc.), linearly distributed over the area, as shown in Figure 2. If the fraction of cellular traffic generated by node *i* is denoted $\omega_{i}$, then $1-\omega_{i}$ is the corresponding fraction of ad hoc traffic. At any time and for any given packet, an IoT device must choose between (i) transmission of the packet to one of its neighbors, as a stepping stone towards the final destination and (ii) sending the packet directly to the base station (cellular network). For instance, a mobile device located far from the base station and attempting to optimize its power consumption may choose to route packets through a multi-hop sequence rather than transmitting them directly to the base station. However, a device located close to the base station may receive a high number of packets to relay to the base station and thus experience a faster battery depletion. The selection strategy in a multi-RAT context can be tuned to reduce this effect, in order to equalize energy depletion across all nodes. The goal consists in optimizing and balancing energy consumption in the network while ensuring that deadlines are met and that data freshness is maintained. This is achieved through the study of two key metrics, namely end-to-end delay and AoI. The main notations and symbols included in this article are listed in Table 2.

For clarification purposes, the model assumptions are summarized below:

1. All nodes are expected to be informed of the success or failure of their transmitted packets. In order to maintain a satisfactory level of reliability permanently, we assume that a packet is re-transmitted (if required) until success or definitive drop;
2. It is expected that each node will have two types of packets to transmit: (1) packets generated by the device itself (queue $Q_{i}$), and (2) packets received from other neighboring devices that must be forwarded until reaching the final destination (queue $F_{i}$);
3. A mobile is capable of transmitting on one interface and receiving on the other. However, it is not able to send an ad hoc and a cellular message on both network cards (no simultaneous transmissions, if we allow parallel selections, we will make multi-homing possible, (i.e., two simultaneous transmissions can be achieved)).
4. It is assumed that the system is not saturated, which entails that the $F_{i}$ and/or $Q_{i}$ queue might be empty at any node *i*.

### 2.2. The Channel Model

In this paper, each IoT device serves both as a relay for forwarding data generated upstream to the next node in a multi-hop chain, and as a cellular transmitter capable of reaching the base station directly. In this context, two distinct channels must be considered, i.e., (1) the ad hoc channel, and (2) the cellular channel.

#### 2.2.1. Ad Hoc Channel

The slotted-Aloha MAC scheme is assumed for all nodes in the ad hoc network, which are also assumed to be perfectly synchronized on certain time slots. Nodes send packets using the following rule. For each time slot, each node independently tosses a coin with a certain bias *p* known as the Aloha medium access probability (MAP). If the result is "heads", it sends the packet in that time slot, otherwise, it does not transmit [29].

We indicate by $N_{i}^{a}$ the average number of transmission attempts, which can be defined as:

(1) $$N_{i}^{a}=\frac{1-{\left(1-\varsigma_{i}\right)}^{K_{i}^{a}}}{\varsigma_{i}},$$

where $K_{i}^{a}$ is the maximum number of transmissions permitted by a mobile *i* per packet.

A transfer from *i* is successful if neither i+1 nor any of its neighbors N(i+1), except *i*, transmits in the same time slot. The success probability $\varsigma_{i}$ for a packet at node *i* in ad hoc network is given by:

(2) $$\varsigma_{i}=q_{i}\prod_{z\in N(i+1)\cup (i+1)\setminus i}\left(1-q_{z}\right),$$

where $q_{i}$ indicates the attempt probability for a packet at node *i*.

#### 2.2.2. Cellular Channel

A Rayleigh channel model is assumed for the cellular channel. The most essential and widely used measure of channel quality in a cellular network is the signal-to-interference-plus-noise ratio (SINR).

The SINR of the IoT device *i* deployed in a fixed location could be written as:

(3) $$\gamma_{i}=\frac{P_{i}\cdot h_{i}}{\sigma^{2}+\sum_{j\neq i}P_{j}\cdot h_{j}\omega_{j}},\;$$

where $P_{i}$ is the transmit power of IoT device *i*, $h_{i}$ refers to her channel gain, which is assumed to follow a Rayleigh distribution and $\sigma^{2}$ is the variance of a Gaussian additive noise.

Here we look at the efficiency function ϕ(γ,L), commonly known as the packet success rate (PSR), for every user who has to send packets of *L* bits each to a base station is denoted as [30]:

(4) $$\phi \left(\gamma_{i},L\right)={\left(1-\xi \left(\gamma_{i}\right)\right)}^{L},\;$$

where *L* is the length of a given packet and ξ(γ) is the bit error rate (BER) from one user to its serving station, which depends on the SINR used. In fact, the expression of BER varies according to the coding and modulation scheme adopted by a user. Our present study is valid for all coding and modulation schemes.

We denote by $N^{c}$ the average number of transmission attempts in a cellular network, which can be expressed as follows:

(5) $$N_{i}^{c}=\frac{1-{\left(1-\phi \left(\gamma_{i},L\right)\right)}^{K_{i}^{c}}}{\phi (\gamma_{i},L)},\;$$

we use $K_{i}^{c}$ to indicate the maximum number of transmissions permitted by a mobile *i* per packet in a cellular network.

### 2.3. Cross-Layer Architecture

**Figure 3:**
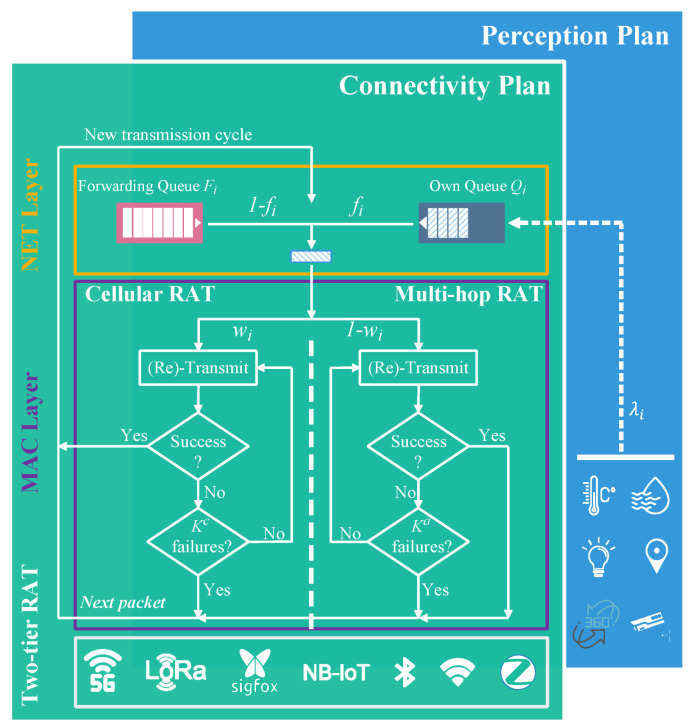
Two-tier IoT network packet transmission cycle and cross-layer flow chart.

Here, a cross-layer architecture is proposed, which takes into account both the network and MAC layer parameters (see Figure 3). Thus, communication and information sharing between separate layers become more efficient and flexible, and offer the possibility of global optimization.

**The network layer** comes first in our cross-layer architecture. It is responsible for defining the source and destination of packets and routing them through the sensor network. It manages two queues: (1) the forwarding queue $F_{i}$, and (2) the queue $Q_{i}$. Queues in the system are assumed to operate with infinite storage capacity, thus avoiding packet loss by overflow. A scheduling method such as first in first out (FIFO) is considered. In addition, a weighted fair queuing (WFQ) is used in the network layer for managing the data transmitted over each cycle. This scheme offers some flexibility and allows QoS support and packet prioritization.

#### 2.3.1. Own Queue $Q_{i}$

This queue handles packets generated by node *i* itself (sensed data in the case of a sensor network), which are transmitted to their final destination (base station) through the network of neighboring sensors, or through the cellular network directly, modeled as an M/G/1 queue, where node *i* opts to transmit from $Q_{i}$ with probability $1-f_{i}$.

#### 2.3.2. Forwarding Queue $F_{i}$

This queue contains packets from other nodes to be forwarded to the base station through one or several hops. It is modeled as a G/G/1 queue, where node *i* decides to forward from $F_{i}$ with probability $f_{i}$. Thanks to this configuration, the nodes benefit from a certain flexibility allowing them to manage the packets transmitted by each node differently from their own packets.

**The MAC layer** establishes the communication media sharing rules for the different IoT devices in the network. Here, we consider a slotted-Aloha MAC protocol. Prior to any transmission attempt, a queue, either $F_{i}$ or $Q_{i}$, is selected. At the beginning of each time slot, a node attempts to gain channel access with random probability $q_{i}$. Then, the head packet from the selected queue is moved from the network layer to the MAC layer where it is transmitted and retransmitted if required, until successful delivery or final drop.

#### 2.3.3. Multi-RAT Support

As mentioned above [4], in the presence of multiple radio interfaces, IoT devices are assumed to be equipped with more than one radio interface and to select the "best" one based on multiple parameters such as user requirements, network capabilities, application properties, etc. In general, every interface has a specific range and cost (energy, economic issues, etc.). A major challenge in multi-RAT networks consists in dynamically selecting the most appropriate RAT in order to address performance goals, such as energy efficiency. Accordingly, an efficient model must be integrated at this decision stage to avoid unnecessary transfer between RATs [31].

#### 2.3.4. Energy Limitation

A major concern in sensor network applications is the capability of operating at ultra-high energy efficiency. Nodes will shut down once their battery is discharged since there is no possibility of recharging them. Indeed, it is assumed that the nodes have no alternate power source such as harvesting, power line, etc. The deployed network must ensure that connectivity is maintained as long as possible, which raises the issue of balancing energy consumption across all nodes. If all nodes in the network consume energy at approximately the same rate, the more central nodes will remain operational and provide forwarding connectivity for a longer time. This leads to more progressive and graceful degradation of the network operation.

### 2.4. Routing within a Two-Tier Network

Our proposed architecture includes two tiers: (1)—the first tier is the proposed multi-hop sensor network, while (2)—the second tier consists of a cellular network.

#### 2.4.1. Tier I: Multi-Hop Network

Sensors are presented as relay nodes, which receive/forward messages from/to their neighbors. We assume static routing, where the IoT device *i* forwards its packets to the mobile device i+1 along a routing chain until the node responsible for relaying to the base station is reached. It is noteworthy that such a multi-hop scheme embodies many well-known benefits, in terms of QoS, generally lower transmission cost, better energy efficiency, longer device lifetime, improved spectrum efficiency/utilization, and higher self-organization capability.

#### 2.4.2. Tier II: Cellular network

Once the packet reaches the sensor node responsible for sending the data to the base station, it is transmitted over the cellular network. We use a multi-RAT network, in which different wireless technologies are combined via separate reliable links (e.g., 5G, LoRa, Sigfox, etc.) for data transfer to the base station.

Finally, these two architectures are unified into a two-tier system to provide an efficient data transfer infrastructure in terms of delay incurred and throughput provided.

Figure 3 depicts an organizational chart that is used to fully understand the connection between the NET and MAC layers for the two-tier IoT network. It is worth mentioning that a transmission cycle comprises a number of time slots that either result in a successful transmission or a failure/drop.

## 3. Average Delay Analysis

Now, we focus our study on the delay, which is a performance metric corresponding to the time needed for a packet to move from source node *s* to the base station, by going either through the multi-hop route or directly through the cellular uplink. We first derive an expression for the entire network, then compute the delay for the cellular and multi-hop sub-systems. Finally, we estimate the arrival and departure rates of our queuing model.

Let $D_{i,j}$ be the cumulative delay that a packet experiences from the moment it is queued at node *i* to the moment it is transmitted over the cellular network by node *j*, given by:

(6) $$D_{i,j}=\sum_{k=i}^{j}D_{k}^{Trans}+D_{k}^{Wait}+D_{k}^{Proc}+D_{k}^{Prop}.$$

For simplification purposes, this paper will only take into account the waiting time and the transmission time, given that both processing time $D_{k}^{Proc}$ and propagation time $D_{k}^{Prop}$ are negligible.

Each packet in the $F_{i}$ or $Q_{i}$ queue, on its way to its neighbor j, has to wait for a certain average time called waiting time ($W_{i}^{F}$ for queue $F_{i}$ and $W_{i}^{Q}$ for $Q_{i}$). Then, in order to complete its transmission, it is directed to the second neighbor, with an ad hoc network service time $t_{i}^{a}$, until node *j* is reached, then it will be transferred to the final destination via the cellular uplink, with a service time corresponding to $t_{i}^{c}$.

**Figure 4:**
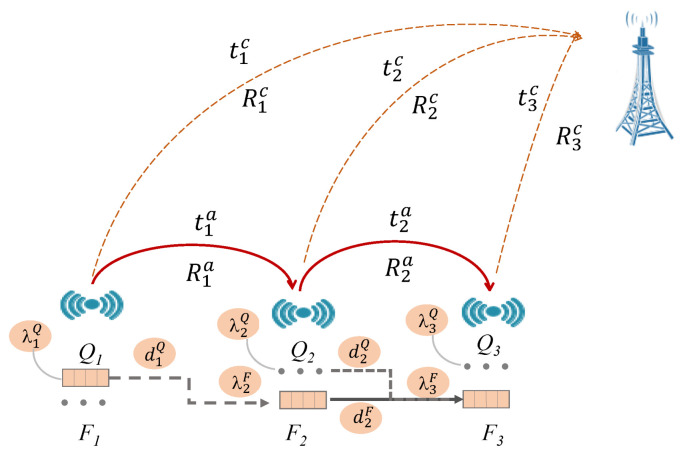
Expected end-to-end delay over two-tier IoT network.

Figure 4 illustrates the expected end-to-end delay in the entire network.

$D_{i,j}$ can be written as follows:

(7) $$D_{i,j}=\left\{\begin{array}{c} \left(t_{i}^{a}+W_{i}^{Q}\right)+\left(t_{j}^{c}+W_{j}^{F}\right)+\sum_{k=i+1}^{j-1}\left(t_{k}^{a}+W_{k}^{F}\right),\;j=i+1,\cdots ,n.\\ W_{i}^{Q}+t_{i}^{c},\;j=i. \end{array}\right.$$

The delay $D_{i}$ experienced at each mobile device *i* is obtained as:

(8) $$D_{i}=\underset{j}{E}\left[D_{i,j}\right]=\sum_{j=i}^{n}D_{i,j}\varphi \left(i,j\right),$$

where $\varphi \left(i,j\right)$ is the probability of sending packets over the multi-hop network from node *i* to node *j*, where the latter will forward the packet to the base station via the cellular uplink, given by:

(9) $$\varphi \left(i,j\right)=\left\{\begin{array}{c} \omega_{j}\prod_{k=i}^{j-1}\left(1-\omega_{k}\right),\;j<n,\\ \prod_{k=i}^{j-1}\left(1-\omega_{k}\right),\;j=n, \end{array}\right.$$

where $\omega_{j}$ denotes the fraction of cellular traffic sent by node *j*.

The average delay generated is obtained as follows:

(10) $$D=\sum_{i}D_{i}\phi_{i},$$

where $\phi_{i}$ is the fraction of the total load contributed by node *i*, expressed as follows:

(11) $$\phi_{i}=\frac{\pi_{i}^{F}+\pi_{i}^{Q}}{\sum_{i}\pi_{i}^{F}+\pi_{i}^{Q}}.$$

Next, we will determine $t_{i}^{c}$, $t_{i}^{a}$, $W_{i}^{F}$ and $W_{i}^{Q}$.

### 3.1. Delay over Cellular Sub-System

Our heterogeneous environment makes multi-RAT systems suitable, where each RAT operates independently from others. Given the possible radio technologies for the tier 2 subsystem, some (e.g., 5G, 6G, NB-IoT,…) are characterized by a deterministic multiple access channel, while others (e.g., LoRa, Sigfox,…) rely on contention to gain access to a shared medium.

The random variable $t_{i}^{c}$ corresponds to the average packet transmission time of user *i* for tier 2, given by:

(12) $$t_{i}^{c}=\left\{\begin{array}{c} N_{i}^{c}\frac{L_{i}}{R_{i}},\;\mathrm{Deterministic}\;\mathrm{multiple}\;\mathrm{access}\;(\mathrm{5G},\;\mathrm{6G},\;\mathrm{NB-IoT},\cdots ),\\ \frac{N_{i}^{c}}{\phi (\gamma_{i},L)},\;\mathrm{Random}\;\mathrm{access}\;\mathrm{through}\;\mathrm{contention}\;(\mathrm{Lora},\;\mathrm{Sigfox},\cdots ), \end{array}\right.$$

where incoming packets are transmitted by user *i* at a rate $R_{i}$ (in bps).

We use $\pi_{i}^{F}$ (resp. $\pi_{i}^{Q}$) to indicate the probability that queue $F_{i}$ (resp. $Q_{i}$) has a packet ready to be transmitted. Moreover, let $\pi_{i,s}^{F}$ be the probability that queue $F_{i}$ has a packet ready to be forwarded. Thus, we have:

(13) $$\pi_{i}^{F}=\sum_{s=1}^{i-1}\pi_{i,s}^{F}.$$

### 3.2. Delay over Multi-Hop Sub-System

We use $t_{i}^{a}$ to represent the average packet transmission time for the ad hoc network (tier 1) at node *i*, given by:

(14) $$t_{i}^{a}=\frac{N_{i}^{a}}{\varsigma_{i}}.$$

### 3.3. Waiting Time

The waiting time in queue $F_{i}$ (resp. $Q_{i}$) is composed of two elements: (1) the queuing time $B_{i}^{F}$ (resp. $B_{i}^{Q}$); and (2) the mean residual service time $R_{i}^{F}$ (resp. $R_{i}^{Q}$). The latter is divided into two terms: (1) the mean residual service time of a tier 2 packet in service $R_{i}^{c}$; (2) the mean residual service time of an ad hoc (tier 1) packet in service $R_{i}^{a}$.

The average waiting time at node *i* in queue $F_{i}$ (resp. $Q_{i}$) is defined as:

(15) $$W_{i}^{F}=R_{i}^{F}+B_{i}^{F},$$

(16) $$W_{i}^{Q}=R_{i}^{Q}+B_{i}^{Q}.$$

#### 3.3.1. Mean residual service time at node *i*:

Any arriving packet should wait until the packet in service is delivered. The latter can be a packet from the $F_{i}$ queue or a packet from the $Q_{i}$ queue, destined directly to the base station (tier 2 network) or next neighbor (*j*) (ad hoc/tier 1 network). The average residual service time observed by a given packet in $F_{i}$ or $Q_{i}$ is denoted:

(17) $$R_{i}^{F}=\omega_{i}R_{i}^{c,F}+\left(1-\omega_{i}\right)R_{i}^{a,F},$$

(18) $$R_{i}^{Q}=\omega_{i}R_{i}^{c,Q}+\left(1-\omega_{i}\right)R_{i}^{a,Q}.$$

Leveraging renewal theory and the method presented in [16], it can be shown that the mean residual service time in $F_{i}$ for cellular network $R_{i}^{c,F}$ and the mean residual service time in $F_{i}$ for ad hoc network $R_{i}^{a,F}$ (resp. $R_{i}^{c,Q}$ and $R_{i}^{a,Q}$) can be expressed as follows:

(19) $$\mathrm{Queue}F:\left\{\begin{array}{c} R_{i}^{c,F}=\frac{t_{i}^{c(2)}}{2t_{i}^{c}}+\frac{1}{2},\\ R_{i}^{a,F}=\frac{t_{i}^{a(2)}}{2t_{i}^{a}}+\frac{1}{2}, \end{array}\right.$$

(20) $$\mathrm{Queue}Q:\left\{\begin{array}{c} R_{i}^{c,Q}=\frac{t_{i}^{c(2)}}{2t_{i}^{c}}+\frac{1}{2},\\ R_{i}^{a,Q}=\frac{t_{i}^{a(2)}}{2t_{i}^{a}}+\frac{1}{2}, \end{array}\right.$$

where $t_{i}^{a(2)}$ and $t_{i}^{c(2)}$ designate the second moment of the service time for the ad hoc and cellular network, respectively, given by [4]:

(21) $$t_{i}^{a(2)}=\frac{N_{i}^{a\left(2\right)}+N_{i}^{a}\left(1-\varsigma_{i}\right)}{\varsigma_{i}^{2}},$$

(22) $$t_{i}^{c(2)}=\left\{\begin{array}{c} N_{i}^{c\left(2\right)}\frac{L_{i}}{R_{i}},\;\mathrm{Deterministic}\;\mathrm{multiple}\;\mathrm{access}\;(\mathrm{5G},\;\mathrm{6G},\;\mathrm{NB-IoT},\cdots ),\\ \frac{N_{i}^{c\left(2\right)}+N_{i}^{c}\left(1-\phi \left(\gamma_{i},L\right)\right)}{\phi {\left(\gamma_{i},L\right)}^{2}},\;\mathrm{Random}\;\mathrm{access}\;\mathrm{through}\;\mathrm{contention}\;(\mathrm{Lora},\;\mathrm{Sigfox},\cdots ), \end{array}\right.$$

where:

(23) $$N_{i}^{a\left(2\right)}=N_{i}^{a}+\frac{2(1-\varsigma_{i})}{\varsigma_{i}^{2}}-\frac{2{\left(1-\varsigma_{i}\right)}^{K_{i}^{a}}\left(K_{i}^{a}-\left(1-\varsigma_{i}\right)\left(K_{i}^{a}-1\right)\right)}{\varsigma_{i}^{2}},$$

(24) $$N_{i}^{c\left(2\right)}=N_{i}^{c}+\frac{2(1-\phi \left(\gamma_{i},L\right))}{\phi {\left(\gamma_{i},L\right)}^{2}}-\frac{2{\left(1-\phi \left(\gamma_{i},L\right)\right)}^{K_{i}^{c}}\left(K_{i}^{c}-\left(1-\phi \left(\gamma_{i},L\right)\right)\left(K_{i}^{c}-1\right)\right)}{\phi {\left(\gamma_{i},L\right)}^{2}}.$$

#### 3.3.2. Queuing time at node *i*:

Once a packet enters the forwarding queue (resp. its own queue), it must wait until the available remaining packets are served before being processed. Once at the head of the forwarding queue (resp. its own queue), it must wait for the packets that will be served before it from its own queue $m_{i}^{Q}$ (resp. the forwarding queue $m_{i}^{F}$). The queuing time in forwarding queue $B_{i}^{F}$ (resp. its own queue $B_{i}^{Q}$) can, therefore, be written as:

(25) $$B_{i}^{F}=m_{i}^{Q}\left(1+m_{i}^{F}\right)\left(\left(1-\omega_{i}\right)t_{i}^{a}+\omega_{i}t_{i}^{c}\right),$$

(26) $$B_{i}^{Q}=m_{i}^{F}\left(1+m_{i}^{Q}\right)\left(\left(1-\omega_{i}\right)t_{i}^{a}+\omega_{i}t_{i}^{c}\right),$$

where $m_{i}^{F}$ is the number of previously entered packets waiting in the forwarding queue. A packet at the top of the forwarding queue (resp. its own queue) ready for transmission must wait a certain number of cycles *X* (random variable) before it can move to the MAC layer. *X* corresponds to the number of cycles required to serve packets from $Q_{i}$ (resp. from $F_{i}$). The probability of waiting *k* cycles is $P\left[X=k\right]={\left(1-f_{i}\right)}^{k}f_{i}$. The expected value of random variable *X* is: $E\left[X\right]≃m_{i}^{Q}≃\frac{1-f_{i}}{f_{i}}$ (resp. $m_{i}^{F}≃\frac{1}{m_{i}^{Q}}≃\frac{f_{i}}{1-f_{i}}$).

Based on Little’s formula $m_{i}^{F}=\lambda_{i,s}^{F}W_{i}^{F}$ for queue $F_{i}$ ($m_{i}^{Q}=\lambda_{i,s}^{Q}W_{i}^{Q}$ for queue $Q_{i}$). The waiting time at node *i* in queue $F_{i}$ (resp. $Q_{i}$) is obtained by using Equation (15) (resp. (16)) and (25) (resp. (26)) as specified below:

(27) $$W_{i}^{F}=\frac{R_{i}^{F}+\left(\left(1-\omega_{i}\right)t_{i}^{a}+\omega_{i}t_{i}^{c}\right)\left(\frac{1-f_{i}}{f_{i}}\right)}{1-\lambda_{i}^{F}\left(\left(1-\omega_{i}\right)t_{i}^{a}+\omega_{i}t_{i}^{c}\right)\left(\frac{1-f_{i}}{f_{i}}\right)},$$

(28) $$W_{i}^{Q}=\frac{R_{i}^{Q}+\left(\left(1-\omega_{i}\right)t_{i}^{a}+\omega_{i}t_{i}^{c}\right)\left(\frac{f_{i}}{1-f_{i}}\right)}{1-\lambda_{i}^{Q}\left(\left(1-\omega_{i}\right)t_{i}^{a}+\omega_{i}t_{i}^{c}\right)\left(\frac{f_{i}}{1-f_{i}}\right)}.$$

### 3.4. Outer Flow

This performance metric measures the rate (per time slot) at which packets are withdrawn from the queues after either a successful transmission or a drop. We identify two independent departure flows: (1) The first flow comprised of packets removed from queue $Q_{i}$, and denoted

(29) $$d_{i}^{Q}=\left(1-\omega_{i}\right)\frac{1-\pi_{i}^{Q}f_{i}}{t_{i}^{a}}+\omega_{i}\frac{1-\pi_{i}^{Q}f_{i}}{t_{i}^{c}},$$

and (2) The second flow comprised of packets removed from queue $F_{i}$, given by:

(30) $$d_{i,s}^{F}=\left\{\begin{array}{c} \frac{\pi_{i,s}^{F}f_{i}}{t_{i}^{a}},\;\mathrm{ad}\;\mathrm{hoc}\;\mathrm{link},\\ \frac{\pi_{i,s}^{F}f_{i}}{t_{i}^{c}},\;\mathrm{Cellular}\mathrm{link}. \end{array}\right.$$

The departure rate $d_{i}^{F}$ experienced at each mobile device *i* is expressed as

(31) $$d_{i}^{F}=\underset{s}{E}\left[d_{i,s}^{F}\right]=\sum_{s}\frac{\pi_{i,s}^{F}f_{i}}{t_{i}^{a}}\left(1-\omega_{i}\right)+\frac{\pi_{i,s}^{F}f_{i}}{t_{i}^{c}}\omega_{i}.$$

### 3.5. Inner Flow

The inner flow is defined as the rate per time slot at which packets arrive at the queues. We identify two independent arrival flows, with (1) the first flow being composed of packets generated by IoT device *i*. Let us assume that packets destined for user *i* will be served with a Poisson distribution whose parameter $\lambda_{i}^{Q}$ corresponds to the average packet arrival rate in its own queue $Q_{i}$, where any packet is composed of *L* bits. The resulting source rate (in bits/second) is therefore indicated by $L\lambda_{i}^{Q}$. The second flow (2) is composed of packets from another neighbor, transmitted via the multi-hop channel. Here, IoT device *i* acts as a cooperative relay to transmit data packets to node *j* responsible for transferring data to the base station.

$\lambda_{i,s}^{F}$ denotes the average packet arrival rate in forwarding queue $F_{i}$ of node *i* from a source *s*, and is expressed as:

(32) $$\lambda_{i,s}^{F}=\left\{\begin{array}{c} 0\;i=s,\\ d_{s}^{Q}\left(1-\pi_{s}^{Q}f_{s}\right)\prod_{z=s}^{i-1}\left(1-\omega_{z}\right)\left(1-{\left(1-\varsigma_{z}\right)}^{K_{z}^{a}}\right).\;\forall i\in N,\;\;\forall s=1,2,\cdots ,i-1. \end{array}\right.$$

**Proof.**  Here we sketch a simplified proof using events decomposition. Let us consider events A and B as follows:

- **Event A:** Traffic generated by mobile device *i* has departed from queue $Q_{s}$;
- **Event B:** All transmissions over successive hops from mobile device *s* to node *i* have been successfully achieved.

We can easily verify that:

$P\left(A\right)=d_{s}^{Q}\left(1-\pi_{s}^{Q}f_{s}\right)$; $P\left(B\right)=\prod_{z=s}^{i-1}\left(1-\omega_{z}\right)\left(1-{\left(1-\varsigma_{z}\right)}^{K_{z}^{a}}\right)$.

As a result, the arrival rate can be expressed as

(33) $$\lambda_{i,s}^{F}=P\left(A\cap B\right),$$

which completes the proof. The total arrival rate at node *i* is then given by:

(34) $$\lambda_{i}^{F}=\sum_{s}\lambda_{i,s}^{F}.$$

□

## 4. Steady State

We estimate in this section the performance metrics in terms of throughput, delay and AoI under steady-state conditions.

In the steady state, the long-term arrival rate is equal to the long-term departure rate. This corresponds to the rate balance Equation (RBE). Thus the $F_{i}$ (resp. $Q_{i}$) queue is stable if its departure rate is at least equal to its arrival rate.

It is written:

(35) $$\left\{\begin{array}{c} \lambda_{i}^{F}=d_{i}^{F},\\ \lambda_{i}^{Q}=d_{i}^{Q}. \end{array}\right.\;\;\forall i\in N.$$

Indeed, given that we have defined the last two metrics, it is possible to determine the expression of the average load $\pi_{i,s}^{F}$ (resp. $\pi_{i}^{Q}$) at each mobile device *i* and for each queue. The RBE results in a linear system, where the queuing system load of $F_{i}$, denoted $\pi^{F}=\left(\pi_{1,s}^{F},\pi_{2,s}^{F},\cdots ,\pi_{i,s}^{F}\right)$, is given by:

(36) $$\pi^{F}=G^{-1}\cdot A,$$

where ***G*** is an I×I matrix and ***A*** is a column vector with the dimensionality I×1.

**Proof.**  Consider the following term obtained by using Equations (31), (34), and (35):

(37) $$\begin{array}{c} \alpha_{i,s}=\frac{d_{s}^{Q}\left(1-\pi_{s}^{Q}f_{s}\right)\prod_{z=s}^{i-1}\left(1-\omega_{z}\right)\left(1-{\left(1-\varsigma_{z}\right)}^{K_{z}^{a}}\right)}{f_{i}\left(\frac{\left(1-\omega_{i}\right)}{t_{i}^{a}}+\frac{\omega_{i}}{t_{i}^{c}}\right)},\;\;\;\;\forall i\in N,\;\;\forall s=1,2,\cdots ,i-1. \end{array}$$

Then:

(38) $$\overset{G}{\overset{︷}{\left(\begin{array}{ccccccc} 1 & 0 & 0 & 0 & 0 & 0 & \cdots \\ 0 & 1 & 0 & 0 & 0 & 0 & \cdots \\ 0 & 0 & 1 & 0 & 0 & 0 & \cdots \\ 0 & 0 & 0 & 1 & 0 & 0 & \cdots \\ 0 & 0 & 0 & 0 & 1 & 0 & \cdots \\ 0 & 0 & 0 & 0 & 0 & 1 & \cdots \\ \vdots & \vdots & \vdots & \vdots & \vdots & \vdots \end{array}\right)}}\cdot \left(\begin{array}{c} \pi_{1,1}^{F}\\ \pi_{2,1}^{F}\\ \pi_{2,2}^{F}\\ \pi_{3,1}^{F}\\ \pi_{3,2}^{F}\\ \pi_{3,3}^{F}\\ \vdots \end{array}\right)=\overset{A}{\overset{︷}{\left(\begin{array}{c} 0\\ \alpha_{2,1}\\ 0\\ \alpha_{3,1}\\ \alpha_{3,2}\\ 0\\ \vdots \end{array}\right)}},$$

we obtain:

(39) $$\pi^{F}=G^{-1}\cdot A,$$

The queuing system load of $Q_{i}$ noted $\pi^{Q}=\left(\pi_{1}^{Q},\pi_{2}^{Q},\cdots ,\pi_{i}^{Q}\right)$ and given by:

(40) $$\pi^{Q}=O^{-1}\cdot Y,$$

where ***O*** is a I×I matrix and ***Y*** is a column vector with dimensionality I×1. □

**Proof.**  Consider the following term obtained by using Equations (29) and (35):

(41) $$\beta_{i}=\frac{1}{f_{i}}\left(1-\frac{\lambda_{i}^{Q}}{\frac{\left(1-\omega_{i}\right)}{t_{i}^{a}}+\frac{\omega_{i}}{t_{i}^{c}}}\right).$$

Then:

(42) $$\overset{O}{\overset{︷}{\left(\begin{array}{ccccccc} 1 & 0 & 0 & 0 & 0 & 0 & \cdots \\ 0 & 1 & 0 & 0 & 0 & 0 & \cdots \\ 0 & 0 & 1 & 0 & 0 & 0 & \cdots \\ 0 & 0 & 0 & 1 & 0 & 0 & \cdots \\ 0 & 0 & 0 & 0 & 1 & 0 & \cdots \\ 0 & 0 & 0 & 0 & 0 & 1 & \cdots \\ \vdots & \vdots & \vdots & \vdots & \vdots & \vdots \end{array}\right)}}\cdot \left(\begin{array}{c} \pi_{1}^{Q}\\ \pi_{2}^{Q}\\ \pi_{3}^{Q}\\ \pi_{4}^{Q}\\ \pi_{5}^{Q}\\ \pi_{6}^{Q}\\ \vdots \end{array}\right)=\overset{Y}{\overset{︷}{\left(\begin{array}{c} \beta_{1}\\ \beta_{2}\\ \beta_{3}\\ \beta_{4}\\ \beta_{5}\\ \beta_{6}\\ \vdots \end{array}\right)}},$$

we obtain:

(43) $$\pi^{Q}=O^{-1}\cdot Y.$$

□

### Age of Information

We are interested in applications where the objective is to continuously communicate the most recently updated state of a time-varying process to a given monitor. As an example, a device sends packets containing a certain state (e.g., sensor data, a list of neighboring nodes) to a network manager on a regular basis to keep the state tracked by the network manager relatively fresh at all times. IoT devices attempt to report their status to the receiver side as soon as possible. The recently proposed AoI metric measures the timeliness and freshness of status updates from various IoT devices at the destination node. It is assumed that time is divided into equal-length slots, and each status update packet is transmitted using exactly one time slot.

**Figure 5:**
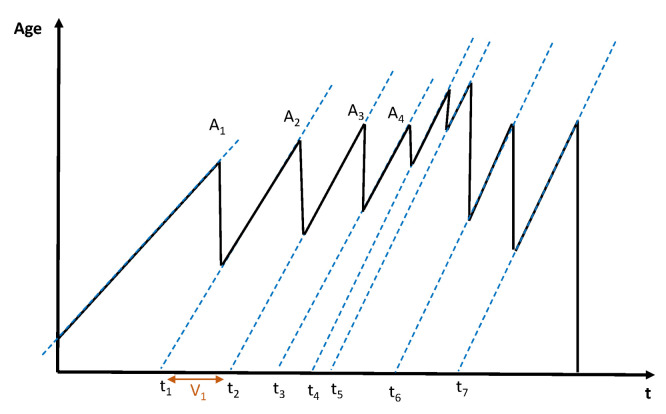
A sample of the evolution of AoI over time.

In Figure 5, we show the evolution of AoI $A_{i}\left(t\right)$ over time where $A_{k}$ indicates the kth peak age, dropping points correspond to the instants when an update packet is received, resulting in a lower age value (i.e., the current time minus the generation time of the new update packet). We can observe from this figure that the AoI is linearly increasing over time and decreasing in case the packets are successfully received.

Given $A_{i}\left(t\right)$, the average age of device *i* can be defined as follows:

(44) $$A_{ave}=\lim_{T\to \infty }\frac{1}{T}\int_{t=0}^{T}A_{i}\left(t\right)dt.$$

Nevertheless, the AoI metric is difficult to analyze. Furthermore, in many systems, it is often the peak state information delay that determines the performance loss. Accordingly, we focus instead on the average peak state age (PAoI) representing the maximum age of the information before a new update is received. As defined in [32], and for a given queuing model, the generalization of PAoI is given by:

(45) $$A_{i}=E\left[V_{i}+W_{i}+t_{i}\right],\;\;\forall i\in N,$$

where $V_{i}$ denotes the inter-arrival time of packets for mobile device *i*, given by:

(46) $$V_{i}=V_{i,s}^{F}+V_{i}^{Q}=\frac{1}{\lambda_{i,s}^{F}}+\frac{1}{\lambda_{i}^{Q}}.$$

For our model, the peak age of information (PAoI) for a packet in the $F_{i}$ (resp. $Q_{i}$) queue from source node *s* to a given neighbor *i* is given by:

(47) $$A_{i,s}^{F}=E\left[V_{s}^{Q}+W_{s}^{Q}+\left(1-\omega_{s}\right)t_{s}^{a}+\sum_{j=s+1}^{i-1}\left(V_{j,s}^{F}+W_{j}^{F}+\left(1-\omega_{j}\right)t_{j}^{a}\right)+V_{i,s}^{F}+W_{i}^{F}+\left(\left(1-\omega_{i}\right)t_{i}^{a}+\omega_{i}t_{i}^{c}\right)\right],\;\;\forall i\in N,$$

(48) $$\begin{array}{c} A_{i}^{Q}=E\left[V_{i}^{Q}+W_{i}^{Q}+\left(\left(1-\omega_{i}\right)t_{i}^{a}+\omega_{i}t_{i}^{c}\right)\right],\;\;\forall i\in N, \end{array}$$

The PAoI obtained at each mobile device *i* is expressed as follows:

(49) $$A_{i}=\underset{\mathrm{Packets}\;\mathrm{recevied}\;\mathrm{from}\;\mathrm{neighbors}}{\underset{︸}{f_{i}\sum_{s=1}^{i-1}A_{i,s}^{F}}}+\overset{\mathrm{Own}\;\mathrm{packets}}{\overset{︷}{\left(1-f_{i}\right)A_{i}^{Q}}}.$$

## 5. Performance Evaluation

This section examines the behavior of the end-to-end delay and the age of information when the fundamental parameters ($f_{i}$, $q_{i}$, $\omega_{i}$, $\lambda_{i}^{Q}$) change. For illustrative purposes, a network of four sensor nodes (n=4) and one base station is considered.

The simulation was carried out under three different scenarios, using MathWorks Matlab R2022b:

1. **Setting 1:** For this first instance, we assumed that the first three nodes (i=1,2,3) have the same fraction of cellular traffic ($\omega_{i}=0.5$), and the node closest to the base station (i=4) needs to relay received packets to the base station, hence we would always retain $\omega_{4}=1$ for all subsequent cases.
2. **Setting 2:** In the second scenario, the node farthest from the base station (i=1) sends all of its packets directly to the base station ($\omega_{1}=1$), the second node (i=2) sends 75% ($\omega_{2}=0.75$) of its packets to the base station and the rest (25% of its packets) to the neighboring node, and third node (i=3) sends 50% ($\omega_{3}=0.5$) of its packets directly to the base station and the rest to the neighboring node. Finally, the last node (i=4) always delivers data with ($\omega_{4}=1$) to the base station.
3. **Setting 3:** For the final case, consider that the node closest to the base station (i=1) sends 25% of its packets to the base station ($\omega_{1}=0.25$), the second node (i=2) sends 50% ($\omega_{2}=0.5$) of its packets to the base station and the rest to the neighboring node, and the third node (i=3) sends 75% ($\omega_{3}=0.75$) of its packets to the base station and the rest to the neighboring node. Finally, the last node (i=4) always provides data to the base station with ($\omega_{4}=1$).

It is worth noting that $f_{1}=0$ as IoT sensor 1 has no predecessor sensor.

### 5.1. Packet Delay

#### 5.1.1. Forwarding Probability $f_{i}$

**Figure 6:**
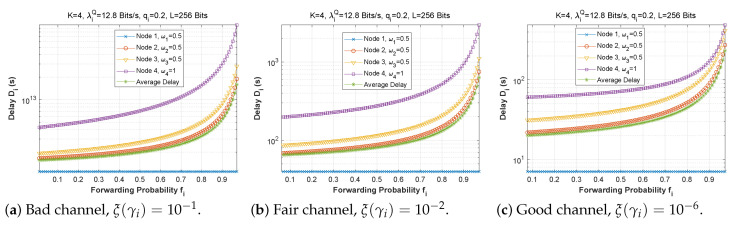
Setting 1: The delay experienced at each mobile device when varying the forwarding probability with the bit error rate $\xi (\gamma_{i})$.

**Figure 7:**
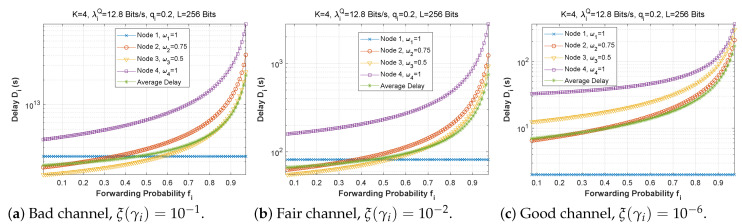
Setting 2: The delay experienced at each mobile device when varying the forwarding probability with the bit error rate $\xi (\gamma_{i})$.

**Figure 8:**
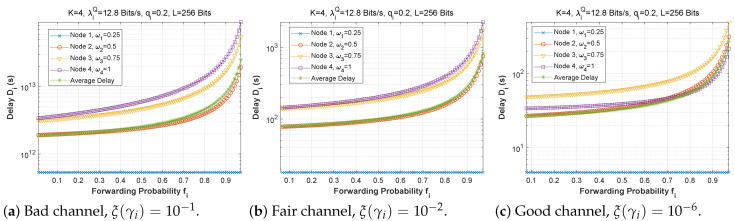
Setting 3: The delay experienced at each mobile device when varying the forwarding probability with the bit error rate $\xi (\gamma_{i})$.

Figure 6, Figure 7 and Figure 8 depict the delay experienced by each mobile device as the forwarding probability changes with bit error rate in the first, second, and third cases, respectively. We can clearly see that using a bad channel ((a), $\xi \left(\gamma_{i}\right)=10^{-1}$) implies a very high delay value, progressing to a lower delay value for a fair channel ((b), $\xi \left(\gamma_{i}\right)=10^{-2}$), and finally reaching a minimal delay by using a good channel ((c),$\xi \left(\gamma_{i}\right)=10^{-6}$). We also notice that the first node does not experience any change in the delay value (a stable delay) because it always has a zero forwarding probability, whereas for the other nodes, the closer we are to the base station, the greater the delay, and an increase in forwarding probability has a direct influence on the obtained latency. This is to be expected, since transmission to neighboring nodes (through the ad hoc network) is preferred, thus nodes closest to the base station will obtain more data packets to transfer. For the third scenario, we notice that node 3 has the longest delay for a good channel, which can be explained by the fact that nodes 1 and 2 transfer more packets to the latter since $w_{1}=0.25$ and $w_{2}=0.5$, and also by the fact that node 3 uses the cellular network ($w_{3}=0.75$) for the transmission of most of the packets received, which further increases the delay.

**Figure 9:**
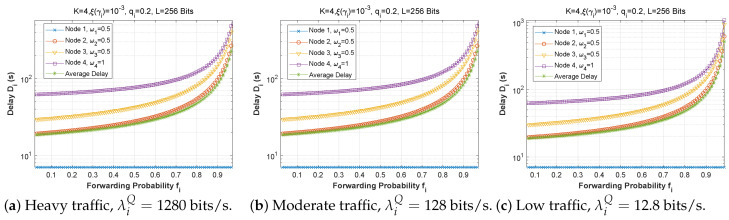
Setting 1: The delay experienced at each mobile device when varying the forwarding probability with the arrival rate $\lambda_{i}^{Q}$.

**Figure 10:**
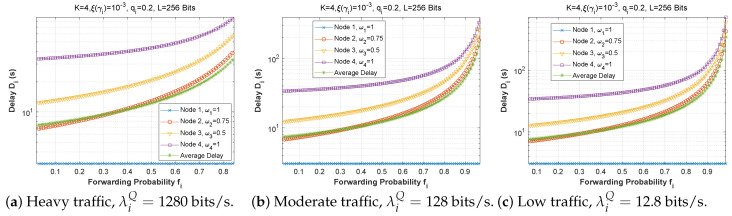
Setting 2: The delay experienced at each mobile device when varying the forwarding probability with the arrival rate $\lambda_{i}^{Q}$.

**Figure 11:**
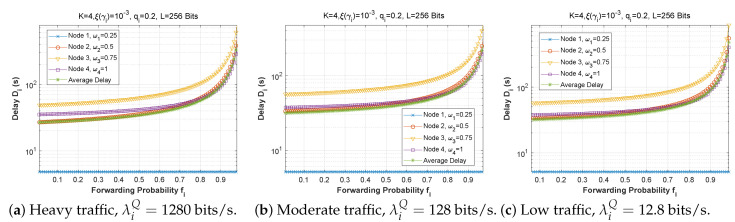
Setting 3: The delay experienced at each mobile device when varying the forwarding probability with the arrival rate $\lambda_{i}^{Q}$.

Figure 9, Figure 10 and Figure 11 demonstrate the delay encountered by each mobile device as a function of the forwarding probability when the arrival rate in the queue varies for the first, second, and third scenarios. We notice that for heavy traffic in scenario 2 (Figure 10a, $\lambda_{i}^{Q}=1280$ bits/s), the delay rises slowly with the increase of the forwarding probability until a maximum is reached when the forwarding probability is close to 1. However, for moderate (Figure 10b, $\lambda_{i}^{Q}=128$ bits/s) and low traffic (Figure 10c, $\lambda_{i}^{Q}=12.8$ bits/s), the delay starts to rise sharply for $f_{i}>0.7$. Furthermore, while the behavior is approximately the same for Figure 10b,c, the curves are slightly lower in the low-traffic case, and in both cases are considerably lower than in Figure 10a. Indeed, as the traffic is reduced, the waiting time in the forwarding queue is likewise reduced. It is noteworthy that in scenarios 1 and 3, the delay curves are nearly the same shape for all three traffic levels, with very slight improvement at reduced traffic.

#### 5.1.2. Attempt Probability $q_{i}$

**Figure 12:**
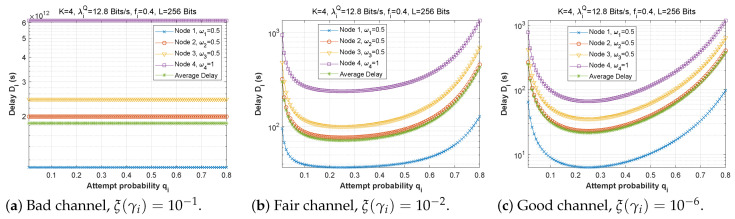
Setting 1: The delay experienced at each mobile device when varying the attempt probability with the bit error rate $\xi (\gamma_{i})$.

**Figure 13:**
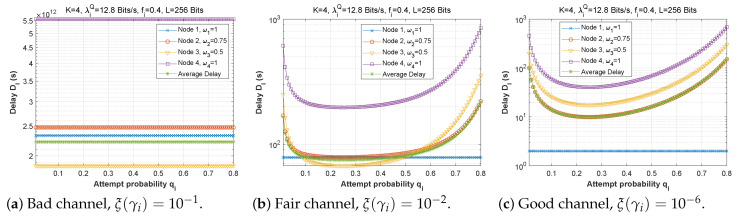
Setting 2: The delay experienced at each mobile device when varying the attempt probability with the bit error rate $\xi (\gamma_{i})$.

**Figure 14:**
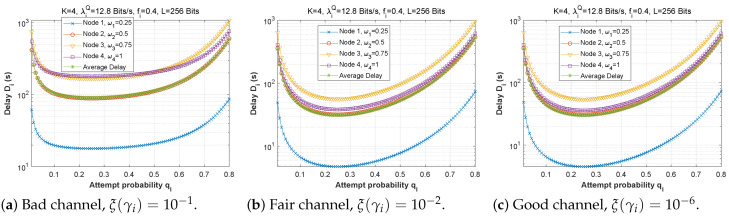
Setting 3: The delay experienced at each mobile device when varying the attempt probability with the bit error rate $\xi (\gamma_{i})$.

Figure 12, Figure 13 and Figure 14 depict how latency varies as a function of attempt probability when the bit error rate changes in the first, second, and third scenarios, respectively. In the first and second scenarios with a bad channel ((a), $\xi \left(\gamma_{i}\right)=10^{-1}$), we can observe that the delay is unstable, reaching very high values of up to $6\times 10^{12}$ s. However, in scenario 3, the delay is more steady, reaching a minimum when the attempt probability is between 0.2 and 0.5.

For the fair ((b), $\xi \left(\gamma_{i}\right)=10^{-2}$) and good ((c), $\xi \left(\gamma_{i}\right)=10^{-6}$) channels, we can see that for very small values of the attempt probability, the system is unstable. Then, for an attempt probability between 0.1 and 0.5, we have a minimal delay. For a value greater than 0.5, the delay begins to increase, which is quite normal given that the system relies heavily on the ad hoc network. We also notice that the node closest to the base station suffers a greater transmission delay than the other nodes, which can be explained by the fact that it receives several packets to transmit, which results in congestion of the queue, and therefore an increase in the transmission delay. For scenario 2, node 1 delivers all of its packets directly to the base station ($w_{1}=0$), which explains its stable latency irrespective of the value of $q_{i}$. In scenario 3, node 3 has the longest delay since it obtains more packets from neighboring nodes ($w_{1}=0.25$ and $w_{2}=0.5$), but node 1 sends most of its packets across the ad hoc network ($w_{1}=0.25$), thus, the change in $q_{i}$ has a significant effect on its transmission delay.

**Figure 15:**
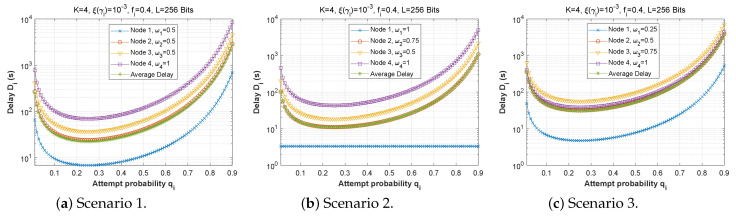
The delay experienced at each mobile device when varying the attempt probability with the arrival rate $\lambda_{i}^{Q}$.

Figure 15 demonstrates the delay encountered by each mobile device as a function of the attempt probability when the arrival rate in the queue is varied (heavy traffic, $\lambda_{i}^{Q}=1280$ bits/s, moderate traffic, $\lambda_{i}^{Q}=128$ bits/s, low traffic, $\lambda_{i}^{Q}=12.8$ bits/s) for the first, second, and third scenarios. We conclude that the fluctuation of $q_{i}$ is unaffected by traffic density since the transmission delay is the same for all traffic categories in all three scenarios.

#### 5.1.3. Fraction of Cellular Traffic $\omega_{i}$

**Figure 16:**
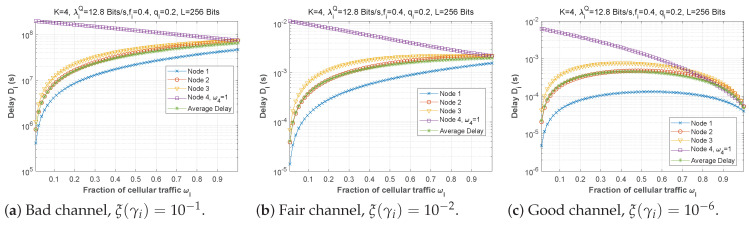
Setting 1: The delay experienced at each mobile device when varying the fraction of cellular traffic with the bit error rate $\xi (\gamma_{i})$.

**Figure 17:**
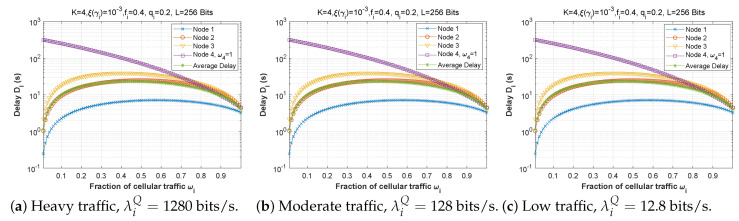
Setting 1: The delay experienced at each mobile device when varying the fraction of cellular traffic with the arrival rate $\lambda_{i}^{Q}$.

Figure 16 demonstrates how delay changes as a function of the fraction of cellular traffic in the first scenario when the bit error rate varies. We can see that the higher the proportion of cellular traffic, the lower the delay for a good channel. Node 4 (the nearest to the base station) always uses a value of $w_{4}=1$, as it only has one option (transmit the packets directly to the base station). The delay is plotted as a function of the fraction of cellular traffic for various regimes in Figure 17. The three subfigures are practically identical, despite the change in the arrival flow in the queue.

#### 5.1.4. Arrival Rate in OWN queue $\lambda_{i}^{Q}$

**Figure 18:**
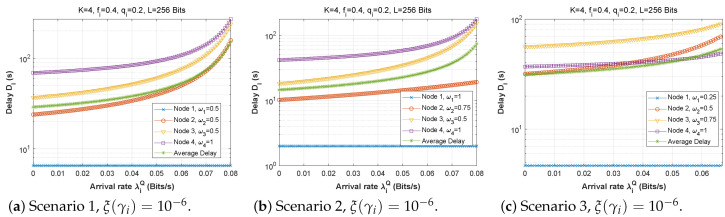
The delay experienced at each mobile device when varying the arrival rate $\lambda_{i}^{Q}$.

We turn now to plot the delay versus the arrival rate for the three scenarios (see Figure 18). For the good channel case, it is shown that the delay increases with an increasing arrival rate for all scenarios. It is apparent that the first node is almost stable, this is because it only carried its own packets.

### 5.2. AoI Simulation

This section examines the behavior of the AoI metric when the fundamental parameters ($f_{i}$, $q_{i}$, $\omega_{i}$, $\lambda_{i}^{Q}$) change.

#### 5.2.1. Forwarding Probability $f_{i}$

**Figure 19:**
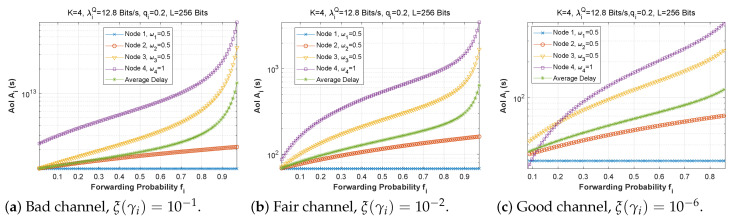
Setting 1: The AoI experienced at each mobile device when varying the forwarding probability with the bit error rate $\xi (\gamma_{i})$.

**Figure 20:**
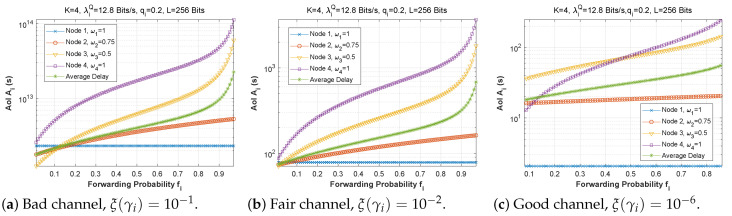
Setting 2: The AoI experienced at each mobile device when varying the forwarding probability with the bit error rate $\xi (\gamma_{i})$.

**Figure 21:**
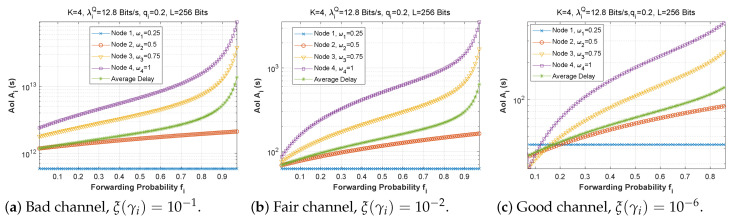
Setting 3: The AoI experienced at each mobile device when varying the forwarding probability with the bit error rate $\xi (\gamma_{i})$.

Figure 19, Figure 20 and Figure 21 depict the AoI as a function of forwarding probability for three values of the bit error rate, and for scenarios 1, 2, and 3. It is obvious that when the forwarding probability is too high, the system suffers from a high AoI and, thus, the AoI per node is high, explaining that an arriving packet cannot be forwarded immediately, due to both a busy MAC layer as well as other packets having priority in the queue. Moreover, passing through multiple nodes (ad hoc network) automatically implies a high AoI.

**Figure 22:**
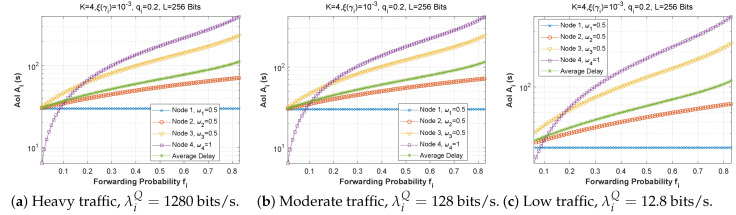
Setting 1: The AoI experienced at each mobile device as a function of forwarding probability $f_{i}$ for various values of the arrival rate $\lambda_{i}^{Q}$.

**Figure 23:**
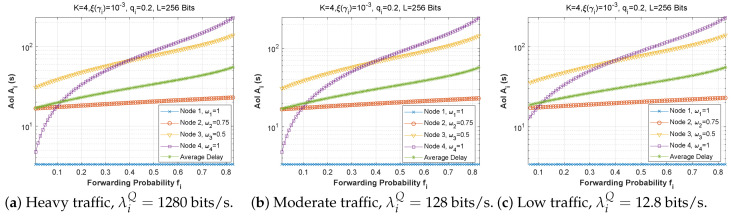
Setting 2: The AoI experienced at each mobile device as a function of forwarding probability $f_{i}$ for various values of the arrival rate $\lambda_{i}^{Q}$.

**Figure 24:**
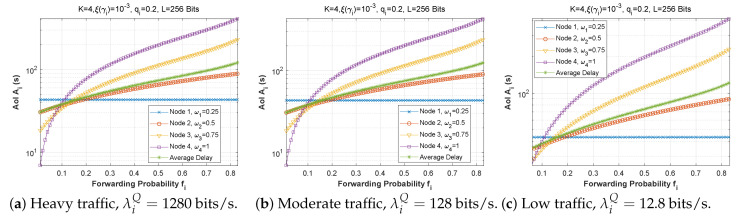
Setting 3: The AoI experienced at each mobile device as a function of forwarding probability $f_{i}$ for various values of the arrival rate $\lambda_{i}^{Q}$.

Figure 22, Figure 23 and Figure 24 depict the AoI as a function of forwarding probability for all three scenarios when the arrival rate in the queue is varied. As observed before, the AoI rises with forwarding probability, and rises all the more quickly the further upstream is the node in the chain (while node 1 has a constant AoI regardless of $f_{i}$). It is noteworthy that the load on the *Q* queue load has little influence on AoI, while the *F* queue load has a significant impact.

#### 5.2.2. Attempt Probability $q_{i}$

**Figure 25:**
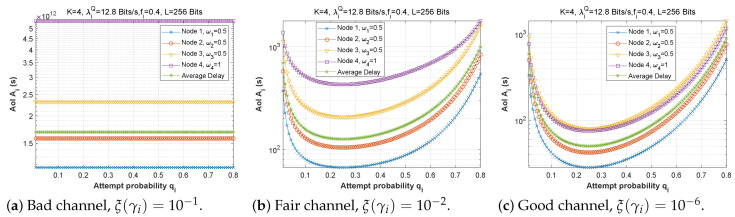
Setting 1: The AoI experienced at each mobile device as a function of attempt probability for various bit error rates $\xi (\gamma_{i})$.

**Figure 26:**
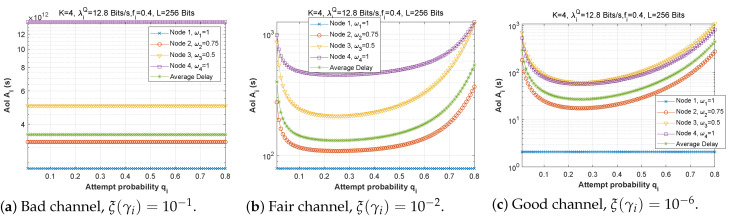
Setting 2: The AoI experienced at each mobile device as a function of attempt probability for various bit error rates $\xi (\gamma_{i})$.

**Figure 27:**
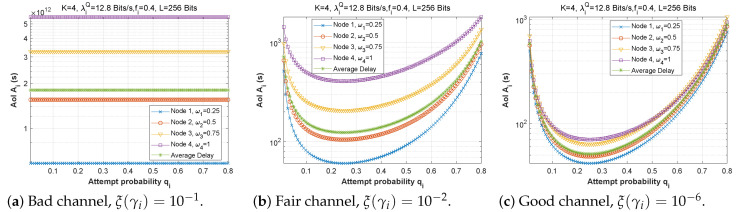
Scenario 3: The AoI experienced at each mobile device as a function of attempt probability for various bit error rates $\xi (\gamma_{i})$.

Figure 25, Figure 26 and Figure 27 show the effect of attempt probability on AoI when the bit error rate is adjusted for the three proposed scenarios. When the attempt probability is too low, the system becomes unstable, and the AoI begins to decrease as the attempt probability decreases, reaching a minimum value when $q_{i}$ is between 0.2 and 0.4. As the attempt probability increases, the queues become more congested, and the AoI increases. Because of the greater attempt probability, more packets will compete for transmission via the ad hoc network. This will tend to overload forwarding queues. Thus, the node farthest from the base station has the largest AoI compared to the node closest to the base station. Since packets in the farthest nodes looking to reach the base station as the target must spend time waiting in each node along the path, and for scenario 2, the first node with $W_{1}=1$ retains a stable AoI.

**Figure 28:**
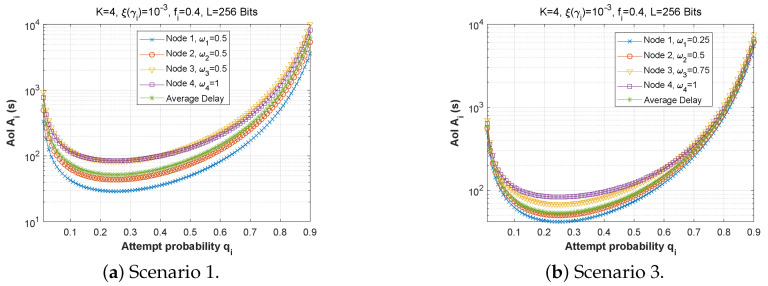
The AoI experienced at each mobile device as a function of attempt probability $q_{i}$ for various arrival rates $\lambda_{i}^{Q}$.

**Figure 29:**
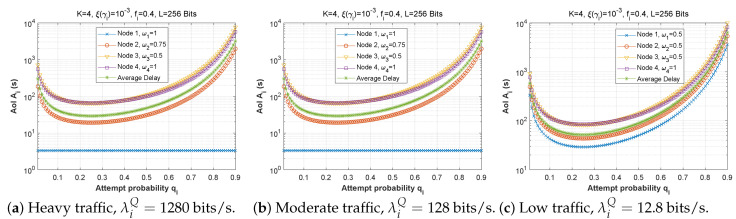
Setting 2: The AoI experienced at each mobile device as a function of attempt probability $q_{i}$ for various arrival rates $\lambda_{i}^{Q}$.

Next, Figure 28 and Figure 29 plot the AoI as a function of attempt probability for various traffic regimes in all three scenarios. Again, it can be observed that the arrival rate in the queue has little impact on the AoI. For scenarios 1 and 3, the subfigures are practically identical, despite the change in the arrival flow in the queue.

#### 5.2.3. Fraction of Cellular Traffic $\omega_{i}$

**Figure 30:**
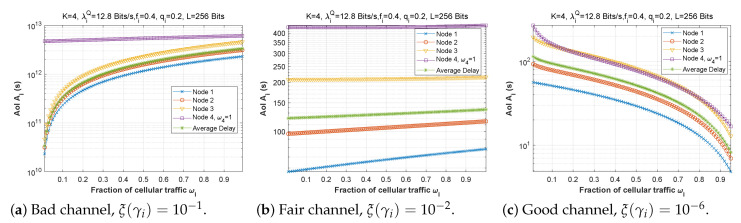
Setting 1: The AoI experienced at each mobile device when varying the fraction of cellular traffic with the bit error rate $\xi (\gamma_{i})$.

For the first scenario, the AoI is plotted as a function of the fraction of cellular traffic in Figure 30. We demonstrate that for a good channel as the fraction of cellular traffic rises, the AoI decreases significantly until it achieves a minimum for $\omega_{i}=0.9$. This decrease in AoI is justified by the fact that nodes send data directly to the base station, implying that packets arrive at their destination faster. Furthermore, the forwarding probability for nodes 1, 2, and 3 is 0.5, resulting in a congested forwarding queue at node 4, explaining its position as the node with the highest AoI.

**Figure 31:**
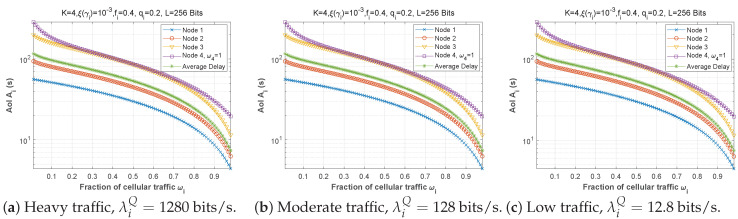
Setting: The AoI experienced at each mobile device when varying the fraction of cellular traffic with the arrival rate $\lambda_{i}^{Q}$.

Next, the AoI is plotted as a function of the fraction of cellular traffic for different values of $\lambda_{i}^{Q}$ in Figure 31. It appears that the system maintains the same behavior regardless of the rate of arrival in the queue *Q*.

#### 5.2.4. Arrival Rate in its Own Queue $\lambda_{i}^{Q}$

**Figure 32:**
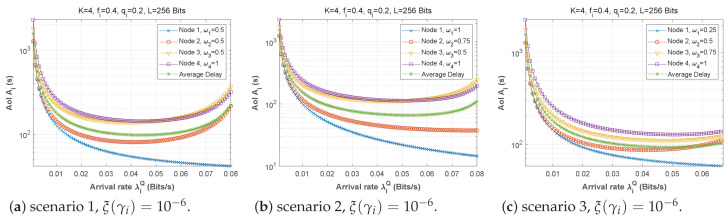
The AoI experienced at each mobile device when varying the arrival rate $\lambda_{i}^{Q}$.

Finally, the AoI is plotted as a function of the arrival rate in queue *Q*, for all three scenarios in Figure 32. In all cases, the AoI is extremely high at low arrival rates. This is because such low levels of traffic imply insufficient status updates at the base station. As $\lambda_{i}^{Q}$ is allowed to increase, the AoI then drops at all nodes until it reaches a minimum value, then rises again, as the queues start to fill up and the system moves toward saturation. The AoI also increases according to the distance of a node from the base station, as this relates to the number of required hops to reach it.

## 6. Concluding Remarks

Multi-hop networks promise to efficiently collect data from IoT devices deployed in a target area as well as relay their data to legacy systems, such as cellular networks. In this paper, we propose a comprehensive theoretical framework for analyzing and understanding the dynamics of such a network. The suggested model is intended to assist mostly in the planning and sizing of an IoT network, as a means of ensuring target/satisfactory performance and effective deployment. We provide a queuing–theory-based model that allows for cross-layered optimization across the APP, NET, MAC, and PHY layers. The suggested model was evaluated using a discrete-event simulation, and it accurately predicts network performance. Our model can measure E2E delay and AoI, making it an excellent choice for evaluating the freshness of information for active streams. It is necessary to examine the impact of forwarding probability, attempt probability, a fraction of cellular traffic, the arrival rate in the queue, and other parameters. The determination of the stability region as a function of these factors constitutes an end result of interest. Many trade-offs have been outlined, as well as a thorough discussion of parameter tuning and network design. This article opens up the way for many exciting areas, including network design and optimal configuration, energy efficiency, wireless energy transfer, flexible infrastructure, etc. Future extensions of this work will examine energy consumption measures to adjust network parameters in order to ensure limited and/or balanced energy consumption.

## Figures and Tables

**Table 2:** Main symbols and their meanings.

Symbol	Meaning
*n*	Number of IoT devices
$\omega_{i}$	Fraction of cellular traffic sent by node *i*
$1-\omega_{i}$	Fraction of traffic sent over the ad hoc link by node *i*
$N_{i}^{a}$	Average number of transmission attempts in ad hoc network
$N_{i}^{c}$	Average number of transmission attempts in cellular network
$K_{i}^{a}$	Maximum number of transmissions permitted by a mobile *i* per packet in ad hoc network
$K_{i}^{c}$	Maximum number of transmissions permitted by a mobile *i* per packet in a cellular network
$\varsigma_{i}$	Success probability for a packet at node *i* in ad hoc network
$q_{i}$	Attempt probability for a packet at node *i*
$\gamma_{i}$	SINR of device *i*
$\phi (\gamma_{i},L)$	Efficiency function
$R_{i}$	Transmission rate (in bps)
*L*	Packet length (in bits)
$\pi_{i}^{F}$	Probability that queue $F_{i}$ has a packet placed at the head of the line
$\pi_{i}^{Q}$	Probability that queue $Q_{i}$ has a packet placed at the head of the line
$f_{i}$	Forwarding probability from queue $F_{i}$
$1-f_{i}$	Forwarding probability from queue $Q_{i}$
$\lambda_{i}^{Q}$	Arrival rate in queue $Q_{i}$
$\lambda_{i}^{F}$	Arrival rate in queue $F_{i}$
$t_{i}^{c}$	Average packet transmission time of user *i* for cellular network
$t_{i}^{a}$	Average packet transmission time for ad hoc network of node *i*
$W_{i}^{F}$	Waiting time in queue $F_{i}$
$W_{i}^{Q}$	Waiting time in queue $Q_{i}$
$B_{i}^{F}$	Queuing time in queue $F_{i}$
$B_{i}^{Q}$	Queuing time in queue $Q_{i}$
$R_{i}^{F}$	Mean residual service time in queue $F_{i}$
$R_{i}^{Q}$	Mean residual service time in queue $Q_{i}$
$R_{i}^{c,F}$	Mean residual service time in queue $F_{i}$ for cellular network
$R_{i}^{a,F}$	Mean residual service time in queue $F_{i}$ for ad hoc network
$R_{i}^{c,Q}$	Mean residual service time in queue $Q_{i}$ for cellular network
$R_{i}^{a,Q}$	Mean residual service time in queue $Q_{i}$ for ad hoc network
$V_{i}$	Inter-arrival time of packets for a mobile device *i*
$A_{i}$	Age of Information for a mobile device *i*
$A_{i}^{F}$	Age of Information for a mobile device *i* in queue $F_{i}$
$A_{i}^{Q}$	Age of Information for a mobile device *i* in queue $Q_{i}$

## Data Availability

Not applicable.
